# Appendicitis in a Newborn: Case Report and Review of the Literature

**DOI:** 10.15388/Amed.2021.29.1.3

**Published:** 2022-01-25

**Authors:** Eleonora Ivanova, Rasa Garunkštienė, Arūnas Liubšys

**Affiliations:** Vilnius University, Faculty of Medicine, Vilnius, Lithuania; Neonatology Centre, Hospital Santaros Klinikos, Vilnius, Lithuania; Clinics of Children Diseases, Institute of Clinical Medicine, Faculty of Medicine, Vilnius University, Vilnius, Lithuania.; Neonatology Centre, Hospital Santaros Klinikos, Vilnius, Lithuania; Clinics of Children Diseases, Institute of Clinical Medicine, Faculty of Medicine, Vilnius University, Vilnius, Lithuania.

**Keywords:** neonatal appendicitis, appendicitis with perforation, NICU

## Abstract

**Background:**

Acute appendicitis in a newborn is rare and may be fatal. The reported incidence is 0,04 % to 0,2 %. Diagnosis remains challenging as the symptoms are undefined.

**Case presentation:**

Here we present a full-term newborn boy of 9 days presenting with malaise, reluctance to feed and subfebrile fever. Over the course of 6 days his condition became worse. The newborn was febrile, passed no stool and his stomach became distended. Perforation due to necrotizing enterocolitis was highly suspected. The diagnosis of acute appendicitis was finalized perioperatively after the perforation and worsening condition made the emergency surgery inevitable. After 16 days of admission the patient was discharged in good condition.

**Conclusion:**

Appendicitis in neonates is a dangerous yet manageable condition. While rare it should be included in differential diagnosis when presented with atypical necrotizing enterocolitis or unexplained peritonitis. Quick and accurate diagnosis may increase survival rates.

## Santrauka.

**Įvadas.:**

Ūminis naujagimių apendicitas yra reta ir pavojinga būklė. Literatūros duomenimis, paplitimas yra 0,04–0,2 procento. Diagnostika sudėtinga, nes naujagimiams būdingi nespecifiniai ir neryškūs simptomai.

**Atvejis.:**

Išnešiotas 9 parų naujagimis atvežtas dėl vangumo, sumažėjusio apetito ir subfebrilaus karščiavimo. Jo būklės dinamika blogėjo, berniukas febriliai karščiavo, nesituštino, pilvas tapo papūstas. Šeštą hospitalizacijos parą įtariant nekrozinį enterokolitą ir galimą perforaciją atlikta skubi laparotomija ir diagnozuotas gangreninis apendicitas su perforacija ir lokaliu peritonitu. Naujagimis po 16 parų hospitalizacijos išrašytas į namus geros būklės.

**Išvada.:**

Apendicitas yra pavojinga liga naujagimystėje, ji turėtų būti diferencijuojama, kai naujagimiui pasireiškia atipinio nekrozinio enterokolito ar lokalaus peritonito požymiai. Greita ir tiksli diagnostika lemia geresnę baigtį.

## Introduction

Neonatal appendicitis is a puzzling finding. The diagnosis is rare and lacks specific diagnostic tools in this age group [1]. Perforation of the appendix is common which leads to higher morbidity and mortality [2]. We present a case of a 9-day-old boy who was diagnosed with perforated appendicitis with localized peritonitis and had a successful operation and discharged in good health.

## Case report

**Figure 1.**
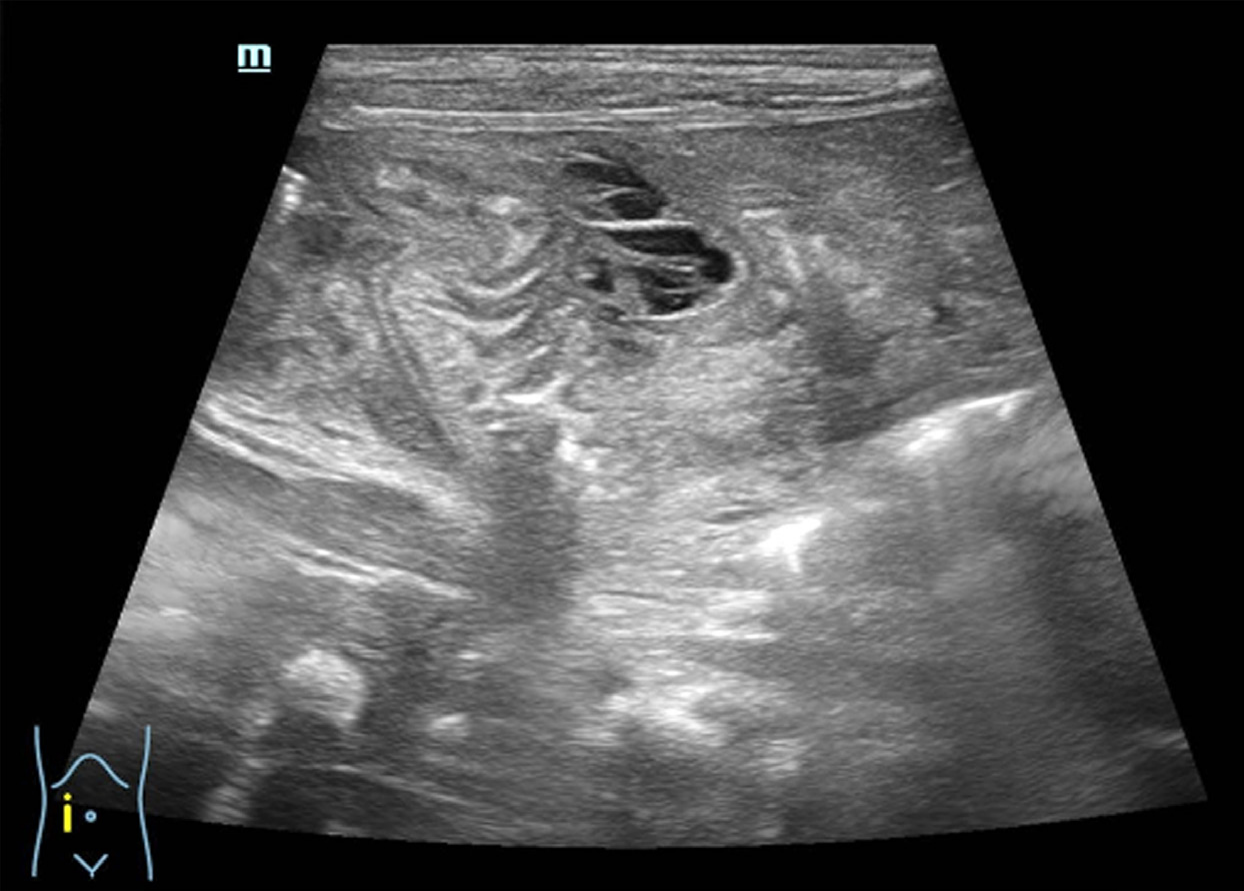
Ultrasonography on third day of admission showing an inflammatory mass

Full-term male neonate was delivered vaginally to 30-year-old mother. His birth weight was 3240 g and Apgar score was 9/10. The neonate was apparently healthy in the first 8 days of life. At the age of 9 days, he was taken to the hospital because of reluctance to feed, moaning and malaise. Examination revealed subfebrile fever, tachycardia 198/min, no respiratory distress. Blood test showed leukocytosis and elevated C reactive protein level of 48 mg/l. The newborn was admitted to neonatal unit with suspicion of perinatal infection and given antibiotic therapy (penicillin and gentamicin). Next day he was feeding better and passed normal stools. On the 3rd day of admission, the newborn became irritable, and his stomach became distended. Ultrasound scan of the abdomen showed an inflammatory mass in the right side of the abdomen, pneumatized segment of the bowel, with collapsed small intestines on the right side compared to the left side (Figure 1).

**Figure 2.**
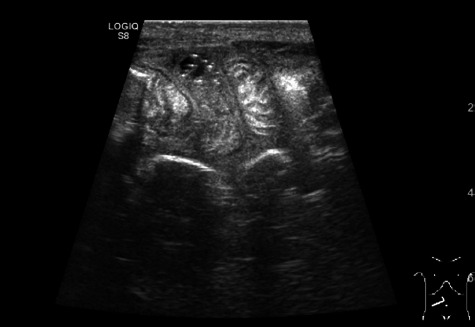
Ultrasonography on fourth day of admission showing oedemic pneumatized bowel

**Figure 3.**
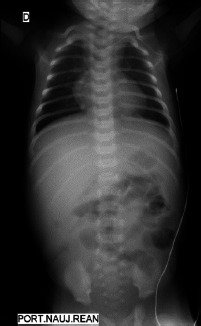
X-ray before operation showing no signs of perforation

Newborn was transferred to the neonatal intensive care unit (NICU). Diagnosis of inflammatory lymphangioma, mesenteric cyst and necrotizing enterocolitis was considered. Abdominal X-rays showed slightly distended bowels. Parenteral nutrition was started along with antibacterial therapy of meropenem while the diagnosis of necrotizing enterocolitis was most likely. Condition remained stable until the 6th day of admission when the newborn became febrile, passed no stools, the ultrasound scan of the abdomen showed fluctuation, oedemic pneumatized bowel walls (Figure 2) while abdominal X-rays remained unremarkable (Figure 3).

**Figure 4.**
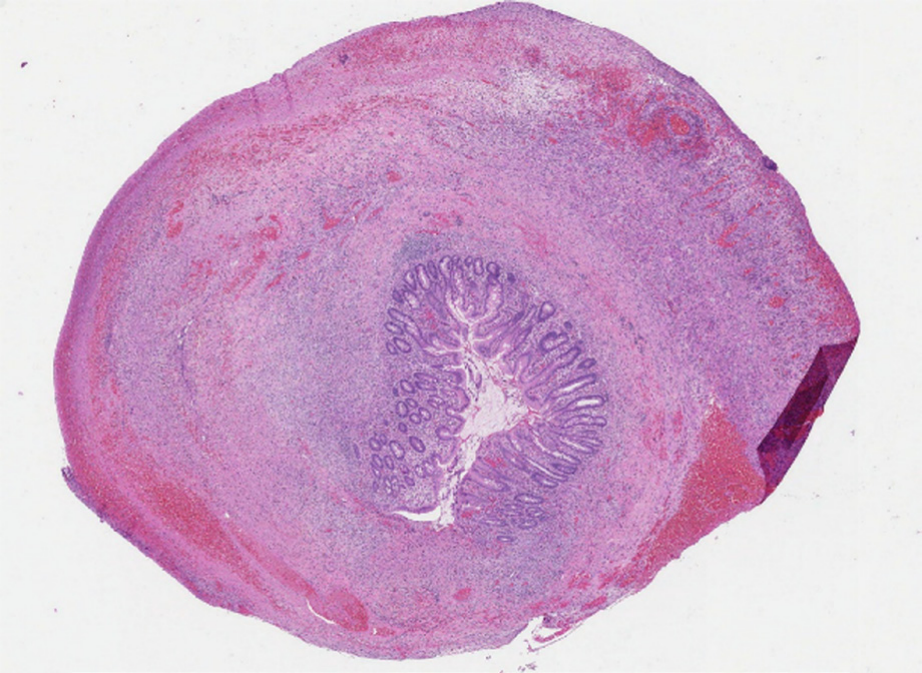
Histology of the surgical specimen

**Figure 5.**
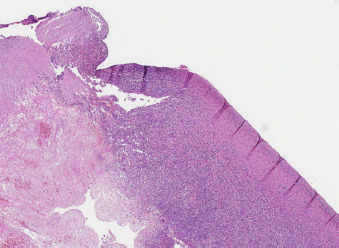
Appendiceal wall necrosis and infiltration

Emergency laparotomy was done with high suspicion of perforated necrotizing enterocolitis. A fibrin infiltrated bowel was found adherent to liver edge with a lot of purulent matter. At the center of the infiltrate a gangrenous appendix was found, and appendectomy with peritoneal lavage was performed. Postoperative period was unremarkable, and the newborn was discharged after 16 days of admission. Histopathological report confirmed acute gangrenous appendicitis with perforation and localized peritonitis and excluded Hirschsprung’s disease (Figure 4, 5).

## Discussion

The most recent literature about neonatal appendicitis mostly reports isolated cases and larger studies are rare. The first case descriptions were autopsy reports and they emerged in the early 20th century [2]. Appendicitis in neonatal population is extremely rare with 141 cases reported since 1905 to 2000 [3]. Incidence is reported to be 0,04% to 0,2% [1,3-5]. Low incidence is attributed to the liquid diet, recumbent position, and funnel shaped appendix with a wide opening into the cecum [2]. These factors lead to a low risk of luminal obstruction, fecaliths [1]. Neonatal appendicitis affects premature and term neonates equally [2,4]. Mortality was reported to be 28% in 2003 [3]. Speculation exists that mortality is actually lower however no comprehensive studies exist [5]. In fact, a 2019 study published 31 cases of neonatal appendicitis and no deaths occurred [4]. Boys are affected in more than half of the cases [2,4].

Incidence of perforation is reported between 69% and 85% [2-4] and seems to be the major point for diagnosis, correlating with increased survival by leading to a faster intervention [2-4]. The anatomically thin appendiceal wall, nondistensible cecum, low incidence of lymphatic hyperplasia and long mesentery is thought to be the reason why the rates of perforation are higher in neonates than older children [1,3]. Time from first symptoms to definitive diagnosis is quicker in newborns with earlier perforation rather than later one (3.3 days vs. 8 days) [2]. If perforation does not occur appendicitis may become chronic and diagnosis may take more than 20 days [6]. In the described case diagnosis was made when the perforation exacerbated the newborns condition. Operation was performed on the 6th day of admission and gangrenous appendix and localized peritonitis found. According to the literature peritonitis develops quickly while the omentum is underdeveloped [1,2,4].

Neonatal appendicitis could be a local form of necrotizing enterocolitis (NEC) which was suspected in the presented case, and difficult to distinguish even after the operation [1,4]. Other conditions associated with neonatal appendicitis is Hirschsprung’s disease and inguinal hernias. Histopathological diagnosis is required to rule out Hirschsprung’s disease. Rectal biopsies may be necessary to rule out this diagnosis while the appendix itself is naturally aganglionic [1]. In our case appendiceal specimen was negative for this condition. Accordingly, the reported incidence of Hirschsprung’s is very low [2]. Amyand hernia – appendicitis found in hernial sac – may be the cause of appendicitis up to ¼ of the cases [3,4]. The presentation is more pronounced with a tender erythematous bulge in the groin, and the mortality is decreased in comparison with the intra-abdominal presentation therefore an early diagnosis and no delay in treatment is expected [1]. Cystic fibrosis was previously attributed to this condition [2]. But as presented in this case the neonate can be previously healthy.

The clinical diagnosis is difficult because symptoms of appendicitis are atypical, other etiologies of abdominal pain are more common and present similarly [1]. This may lead to misdiagnosis and mismanagement [4]. Neonatal appendicitis usually presents with anorexia, irritability, abdominal distension, fever, elevated C reactive protein [1-5]. Bilious emesis, diarrhea, hematochezia and elevated white blood count is possible [1,3,6]. The most common initial diagnosis for these symptoms is NEC and inguinal hernia. Appendicitis is suspected infrequently [4].

The diagnostic tools for neonatal appendicitis are unspecific. Radiographs are usually done but are only useful at the stage of perforation [7]. However, in the presented case the radiographs were unremarkable throughout, even after the suspected time of perforation. Computed tomography is not recommended but may show more specific findings [7]. The abdominal ultrasound is a useful tool and can show intra-abdominal abscess, localized changes indicating a collection in the right iliac fossa. However, the appendix in a higher position near the liver edge may be unnoticed. Furthermore, after perforation occurs the accompanying edema with fluid makes the appendix difficult to visualize. This should in turn increase the suspicion of appendicitis. Evaluation of nearby structures for inflammatory masses and stiff, fixed loops of adjacent bowels should follow [1]. Just like in the presented case ultrasonography can raise suspicion for cystic lesions of lymphangiomas, thus making the differential diagnosis more challenging [2].

Management of appendicitis in neonate consists of typical appendectomy and peritoneal lavage [3]. The operation can be performed through laparotomy or with laparoscopic approach [1]. The aim should be to have the surgical intervention early and less invasively [1,3].

## Conclusion

Appendicitis in neonates is a rare condition with a challenging diagnosis and high mortality. However, this diagnosis should come to mind when presented with atypical NEC or unexplained peritonitis. Quick and accurate diagnosis may increase survival rates.

## Declaration of interests

The authors declare that they have no conflict of interest.

## Informed consent

Written informed consent was obtained from the legal guardians of the patient for the purposes of publication of this case report and any of accompanying images.
